# Serum Protein Electrophoresis and the Albumin-to-Globulin Ratio in the Differential Diagnosis of Minimal Change Disease and Focal Segmental Glomerulosclerosis

**DOI:** 10.3390/biomedicines14030720

**Published:** 2026-03-20

**Authors:** László Bitó, Tamás Lantos, Krisztina Jost, Amir Reza Manafzadeh, Béla Iványi, Levente Kuthi

**Affiliations:** 1 Department of Internal Medicine, Albert Szent-Györgyi Medical School, University of Szeged, 6722 Szeged, Hungary; manafzadeh.amir.reza@med.u-szeged.hu; 2 Department of Medical Physics and Informatics, Albert Szent-Györgyi Medical School, University of Szeged, 6720 Szeged, Hungary; lantos.tamas@med.u-szeged.hu; 3 Institute of Laboratory Medicine, Albert Szent-Györgyi Medical School, University of Szeged, 6725 Szeged, Hungary; jost.krisztina@med.u-szeged.hu; 4 Department of Pathology, Albert Szent-Györgyi Medical School, University of Szeged, 6725 Szeged, Hungary; ivanyi.bela@med.u-szeged.hu; 5 Department of Surgical and Molecular Pathology, Tumor Pathology Center, National Institute of Oncology, 1122 Budapest, Hungary; kuthi.levente@oncol.hu; 6 Department of Pathology and Experimental Cancer Research, Semmelweis University, 1085 Budapest, Hungary; 7 HUN-REN-ONKOL-TTK-HCEMM Oncogenomics Research Group, National Institute of Oncology, 1122 Budapest, Hungary

**Keywords:** minimal change disease, focal segmental glomerulosclerosis, serum protein electrophoresis, albumin-to-globulin ratio, nephrotic syndrome, biomarker

## Abstract

**Background/Objectives:** Differentiating minimal change disease (MCD) from focal segmental glomerulosclerosis (FSGS) remains a diagnostic challenge. We hypothesised that differences in glomerular protein selectivity could translate into distinct serum protein electrophoresis (SPEP) profiles, particularly in severe nephrotic syndrome. **Methods:** We retrospectively analysed SPEP profiles of adults with biopsy-proven MCD (*n* = 27), primary FSGS (*n* = 27), and secondary FSGS (*n* = 20). Diagnoses were established according to KDIGO guidelines and the Mayo Clinic classification. A severe subgroup was defined by a relative albumin fraction <40% to evaluate patterns in marked hypoalbuminaemia. **Results:** Secondary FSGS demonstrated significantly higher albumin-to-globulin (A/G) ratios compared with immune-mediated podocytopathies (MCD and primary FSGS), yielding excellent discrimination (AUC > 0.98). In contrast, discriminatory performance between MCD and primary FSGS in the overall cohort was limited (AUC = 0.657). However, within the severe subgroup, the A/G ratio provided clinically meaningful separation (AUC = 0.787). An A/G ratio > 0.49 identified primary FSGS with 86.7% sensitivity and 81.2% specificity. Correlation analysis revealed a strong inverse association between albumin and α2-globulin fractions in immune-mediated podocytopathies (*ρ* < −0.8), whereas this relationship was attenuated in secondary FSGS (*ρ* = −0.57). **Conclusions:** The A/G ratio may represent a practical adjunctive biomarker in the evaluation of podocytopathies. Values > 1.0 strongly favour secondary FSGS, while markedly reduced ratios in severe nephrosis are characteristic of MCD. These findings suggest that differences in glomerular selectivity and the hepatic compensatory response are reflected in routine electrophoretic profiles.

## 1. Introduction

Podocytopathies, including minimal change disease (MCD) and focal segmental glomerulosclerosis (FSGS), represent major causes of idiopathic nephrotic syndrome in adults [1]. Although membranous nephropathy remains prevalent, epidemiological data from large biopsy registries indicate a rising incidence of FSGS, which has become the leading cause of primary nephrotic syndrome in several multiethnic populations [1].

Distinguishing MCD from FSGS remains a clinical challenge [2]. Although the two conditions often present with similar clinical features characterised by heavy proteinuria, their underlying pathobiology differs substantially. In MCD, injury is characterised by diffuse podocyte foot process effacement without significant podocyte loss, thereby allowing potential reversibility [3,4]. In contrast, FSGS is defined by podocyte depletion and irreversible segmental scarring, reflecting permanent structural damage to the glomerular filtration barrier [5,6].

These distinct pathological processes result in differences in glomerular permeability. MCD typically produces highly selective proteinuria predominantly involving albumin, whereas FSGS is associated with nonselective proteinuria characterised by leakage of higher-molecular-weight proteins. [7]. Although renal biopsy remains the diagnostic gold standard, its diagnostic accuracy is limited by sampling variability, particularly in FSGS, where lesions are focal and segmental [3,8]. Moreover, distinguishing primary from secondary FSGS has direct therapeutic implications, as inappropriate immunosuppression may expose patients to unnecessary risk [9].

Serum protein electrophoresis (SPEP) is routinely performed in the evaluation of nephrotic syndrome. Beyond its established role in detecting monoclonal gammopathies, SPEP reveals a characteristic “nephrotic pattern” marked by hypoalbuminaemia and relative elevation of α2 and β fractions [10]. These changes reflect both urinary protein loss and compensatory hepatic synthesis [11,12]. While this pattern is generally regarded as a uniform response to nephrotic-range proteinuria [13], it remains unclear whether subtle differences in electrophoretic profiles reflect the selectivity of the underlying glomerular injury.

Albumin contributes disproportionately to plasma oncotic pressure due to its abundance and molecular characteristics [14]. Selective albumin loss may therefore trigger distinct systemic responses compared with non-selective proteinuria [15]. We hypothesised that differences in glomerular size-selectivity between MCD and FSGS could translate into measurable differences in serum protein fractions, particularly in the albumin-to-globulin (A/G) ratio.

This study aimed to compare SPEP profiles in patients with biopsy-proven MCD and FSGS and to determine whether electrophoretic patterns can serve as a non-invasive adjunct in differentiating podocytopathies. To our knowledge, a systematic comparative analysis of SPEP patterns across these podocytopathies has not previously been reported in the English-language literature.

## 2. Materials and Methods

### 2.1. Study Population

The study was approved by the Regional Human Biomedical Research Ethics Committee (Licence No. 26/2024-SZTE RKEB). We retrospectively analysed 74 adult patients with biopsy-proven podocytopathies treated at the University of Szeged between 2009 and 2025. The final study cohort comprised patients with MCD (*n* = 27), primary FSGS (*n* = 27), and secondary FSGS (*n* = 20). Within the MCD group, a secondary aetiology was identified in 18.5% of patients. Inclusion criteria were a definitive histological diagnosis of MCD or FSGS and the availability of complete clinical and laboratory data from the peri-biopsy period. To minimise systemic confounders that might affect SPEP profiles, strict exclusion criteria were applied. Patients with monoclonal gammopathy, liver cirrhosis, or prior immunosuppressive therapy before sampling were excluded. Furthermore, patients with a documented history of active infectious diseases (e.g., HIV, hepatitis B or C) and those with severe secondary metabolic nephropathies (e.g., advanced diabetic nephropathy) were also excluded from the analysis. Because the primary aim of this study was the differential diagnosis among specific pathological entities presenting with nephrotic syndrome, a healthy control (negative) group was not included. Diagnoses were established based on clinical and histopathological findings. The kidney samples were obtained by an ultrasound-guided percutaneous biopsy. All samples were evaluated by light microscopy using special staining, direct immunofluorescence (IF) on frozen sections (FITC-conjugated antibodies to IgG, IgA, IgM, C3, C1q, kappa, lambda, and fibrinogen [Dako, Glostrup, Denmark]), and electron microscopy (EM).

MCD was diagnosed in patients with nephrotic syndrome who exhibited minimal or no abnormalities on light microscopy or IF, and diffuse podocyte foot process effacement (≥80% of the capillary circumference) was observed on EM [16].

The clinicopathologic approach of the Mayo Clinic was used to differentiate primary and secondary FSGS. Primary FSGS was diagnosed in patients with massive proteinuria or nephrotic syndrome when segmental sclerosis was identified in at least one glomerulus; IF microscopy excluded immune complex deposition, EM demonstrated diffuse podocyte foot process effacement, and secondary causes of FSGS, such as long-standing hypertension, adaptive-functional responses, malignancy, drug exposure, or viral infection, were clinically excluded [17].

Secondary FSGS was diagnosed when a proteinuric patient exhibited an FSGS lesion on light microscopy; there were no immune complexes on IF, EM revealed segmental podocyte foot process effacement, and secondary causes of FSGS were present clinically [9].

For analytical purposes, primary FSGS and the entire MCD cohort (including secondary forms) were collectively referred to as immune-mediated podocytopathies [18], consistent with current pathophysiological concepts.

### 2.2. Laboratory Analysis

Serum samples were obtained at the time of renal biopsy or during the immediate peri-biopsy period, before initiation of disease-specific immunosuppressive therapy.

SPEP was performed using a Sebia Hydrasys 2 system (Sebia, Lisses, France) with agarose gel (Sebia Hydragel B1–B2), and standard fractions were quantified (albumin, α1, α2, β, and γ) [10].

The A/G ratio was calculated from relative electrophoretic fractions as:

$$A/G\;\mathrm{ratio}=\frac{\mathrm{Albumin}\;(\%)}{\alpha 1+\alpha 2+\beta +\gamma }$$

where globulins represent the sum of the α1, α2, β, and γ fractions.

### 2.3. Definition of Severe Subgroup

Patients were stratified into a severe subgroup defined by a relative albumin fraction <40%. This threshold corresponds approximately to the classical nephrotic range of absolute serum albumin (<25 g/L; <30 g/L in contemporary guidelines) [19]. The relative albumin fraction was selected to minimise the influence of volume status and haemodilution, which may confound absolute albumin concentrations [20].

This stratification enabled comparison among patients with similarly severe hypoalbuminaemia.

### 2.4. Statistical Analysis

Normality was assessed using histograms and the Shapiro–Wilk test. Normally distributed continuous variables were analysed using the independent samples *t*-test or one-way ANOVA with Tukey’s HSD post hoc test and are presented as mean ± SD. Non-normally distributed variables were analysed using the Mann–Whitney *U* test or the Kruskal–Wallis test with Dwass–Steel–Critchlow–Fligner post hoc comparisons and are presented as median (interquartile range, IQR). Associations were assessed using Spearman’s rank correlation coefficient (*ρ*). Categorical variables were compared using the chi-squared test. Diagnostic performance was evaluated using receiver operating characteristic (ROC) curves, and the area under the curve (AUC) was calculated. All tests were two-sided, with *p* < 0.05 considered statistically significant. All analyses were performed using R version 4.4.1 (R Foundation for Statistical Computing, Vienna, Austria).

## 3. Results

### 3.1. Baseline Characteristics of the Study Population

Baseline demographic and laboratory characteristics are summarised in Table 1. No statistically significant differences were observed among the three groups with respect to age (*p* = 0.114) or sex distribution (*p* = 0.251), indicating comparable demographic comparability.

Marked differences were observed in laboratory profiles. Patients with MCD and primary FSGS demonstrated a phenotype consistent with severe nephrotic syndrome, characterised by pronounced hypoalbuminaemia and elevated α2- and β-globulin fractions. In contrast, patients with secondary FSGS exhibited significantly higher median relative albumin fractions (60.3% vs. approximately 36–37% in MCD and primary FSGS; *p* < 0.001) and a comparatively preserved electrophoretic profile, consistent with the absence of overt nephrotic syndrome.

Importantly, no statistically significant differences were detected in electrophoretic patterns between patients with idiopathic MCD and those with identifiable secondary causes. Given this phenotypic overlap, these subgroups were pooled for subsequent analyses. Furthermore, strict exclusion criteria were applied to minimise potential confounders affecting the A/G ratio. In particular, monoclonal gammopathy (paraproteinaemia) was absent in all patients, ensuring that the observed electrophoretic alterations reflected podocytopathy-associated protein distribution.

### 3.2. The ‘Quiescent Liver’: Identifying Secondary FSGS

**Figure 1:**
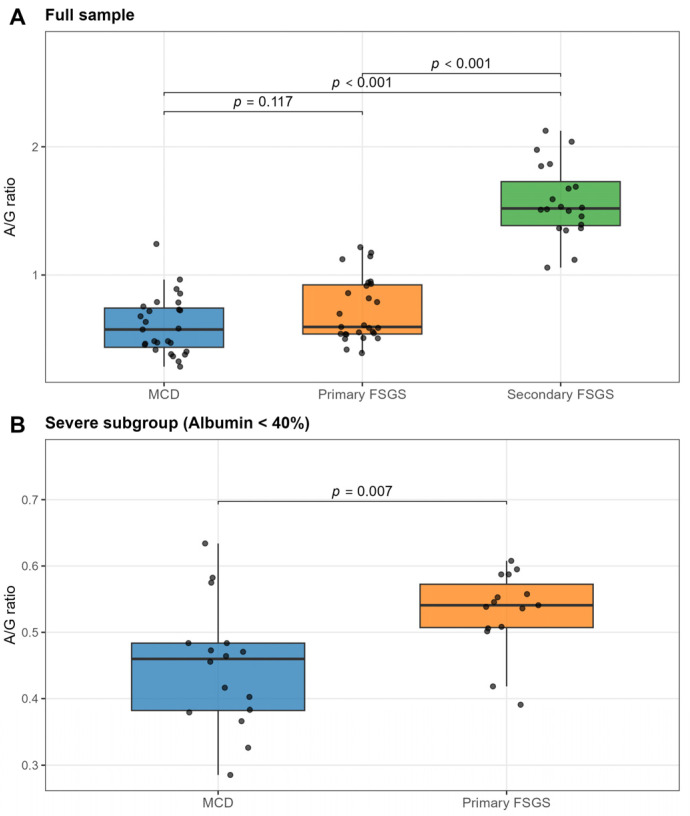
Comparison of albumin-to-globulin (A/G) ratios across podocytopathy subtypes. (**A**) Boxplots of the A/G ratio in the full study cohort. Secondary FSGS (green) demonstrates a significantly higher A/G ratio compared with primary podocytopathies, whereas no statistically significant difference is observed between MCD and primary FSGS. (**B**) Analysis restricted to the severe subgroup (relative albumin < 40%). In this subgroup, a statistically significant difference is observed between MCD and primary FSGS, with a lower A/G ratio in MCD. Horizontal lines represent medians; box boundaries indicate interquartile ranges (IQRs). Here, *p*-values were calculated using the Kruskal–Wallis test (**A**) and Mann–Whitney *U* test (**B**).

**Figure 2:**
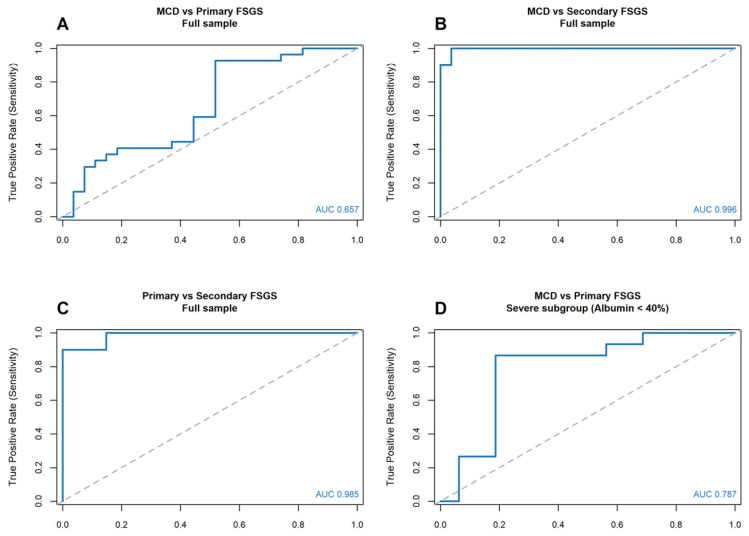
Diagnostic performance of the albumin-to-globulin (A/G) ratio. ROC curves illustrating the discriminatory capacity of the A/G ratio across different comparisons. (**A**) Full cohort comparison between MCD and primary FSGS, demonstrating limited discrimination (AUC = 0.657). (**B**,**C**) Differentiation of secondary FSGS from immune-mediated podocytopathies. The A/G ratio shows excellent discriminatory performance for distinguishing secondary FSGS from MCD (panel B, AUC = 0.996) and from primary FSGS (panel C, AUC = 0.985). (**D**) Severe subgroup (relative albumin fraction < 40%) comparing MCD and primary FSGS, demonstrating improved discrimination (AUC = 0.787). The solid blue line represents the receiver operating characteristic (ROC) curve for the A/G ratio, while the dashed diagonal line indicates the reference line of no discrimination (random chance).

Secondary FSGS exhibited a markedly distinct serum protein profile compared with immune-mediated podocytopathies, as illustrated by both boxplot analysis (Figure 1A) and ROC analysis (Figure 2B,C).

**Figure 3:**
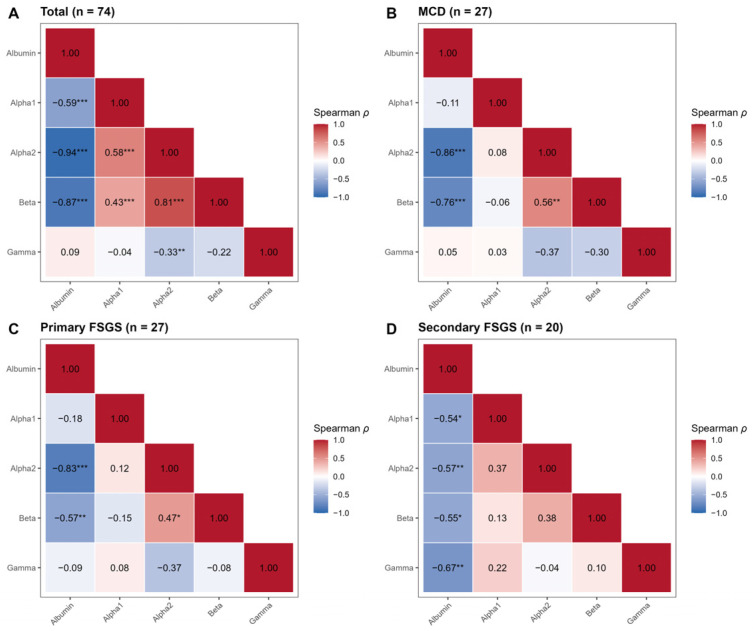
Spearman correlation matrices of electrophoretic fractions. Heatmaps illustrating pairwise Spearman correlation coefficients (*ρ*) between electrophoretic protein fractions (Albumin, α1-, α2-, β-, γ-globulin). Red indicates positive correlation and blue indicates negative correlation. (**A**) Total cohort. (**B**) MCD. (**C**) Primary FSGS. (**D**) Secondary FSGS. Values inside the cells represent Spearman’s *ρ*. Statistical significance is indicated as follows: * *p* < 0.05, ** *p* < 0.01, *** *p* < 0.001.

The median A/G ratio was significantly higher in secondary FSGS (1.52, IQR 1.39–1.73) than in MCD (0.58, IQR 0.44–0.74) and primary FSGS (0.60, IQR 0.54–0.92) (*p* < 0.001 for both comparisons). Consequently, the A/G ratio demonstrated excellent discriminatory ability for identifying secondary FSGS. An A/G ratio > 1.01 effectively excluded MCD (sensitivity 100%, specificity 96.3%; AUC = 0.996) and robustly differentiated secondary from primary FSGS (AUC = 0.985). These findings indicate that preservation of the A/G ratio is characteristic of secondary FSGS and contrasts with the pronounced electrophoretic alterations observed in immune-mediated podocytopathies. This distinct pathophysiological pattern is further reflected in the correlation matrices (Figure 3). Whereas immune-mediated podocytopathies exhibited a strong inverse association between albumin fraction and α2-globulin fraction, secondary FSGS showed a significantly weaker correlation (*ρ* = −0.57, *p* = 0.009; Figure 3D). This relative attenuation suggests a reduced coupling between albumin depletion and compensatory α2-globulin increase in secondary FSGS.

### 3.3. MCD vs. Primary FSGS: The ‘Selective Retention’ Effect

In the overall study population, discrimination between MCD and primary FSGS was modest. As shown in Figure 1A and Figure 2A, there was substantial overlap in A/G ratios between the two groups, and the difference did not reach statistical significance (*p* = 0.117). Accordingly, diagnostic performance in the full cohort was modest (AUC = 0.657).

This overlap likely reflects the inclusion of patients with milder disease activity or partial remission, in whom electrophoretic alterations may be attenuated and therefore insufficient to produce distinct biochemical separation between the two entities.

However, within the severe subgroup (defined as relative albumin fraction < 40%), the biological distinction became more pronounced and clinically relevant (Figure 1B and Figure 2D).

In contrast to the full cohort, the severe subgroup (relative albumin fraction < 40%) demonstrated a statistically significant difference in the A/G ratio between MCD and primary FSGS (*p* = 0.007). Within this subgroup, the A/G ratio showed moderate-to-good discriminatory performance, with an AUC of 0.787. From a clinical perspective, an A/G cut-off >0.494 identified primary FSGS with a sensitivity of 86.7% and a specificity of 81.2%, indicating clinically meaningful separation in the context of advanced nephrotic syndrome. Severe MCD was characterised by a markedly reduced A/G ratio (median < 0.49), whereas primary FSGS exhibited relatively higher values. This pattern suggests differential handling of globulin fractions under conditions of profound hypoalbuminaemia. One possible explanation is that in fully developed nephrotic states, compensatory increases in α2- and other globulins are more prominently retained in MCD, whereas primary FSGS may allow relatively greater globulin loss. However, this mechanistic interpretation remains inferential and warrants further investigation.

### 3.4. Correlation Analysis: The Regulation of Hepatic Response

We examined the association between relative albumin fraction and α2-globulin fraction using Spearman’s rank correlation to characterise the consistency of the compensatory response of the liver across disease subtypes (Figure 3).

MCD demonstrated a very strong inverse correlation (*ρ* = −0.86, *p* < 0.001) (Figure 3B), indicating a tightly coupled relationship between albumin depletion and α2-globulin elevation. This pattern suggests a highly consistent compensatory response in severe nephrotic states associated with MCD. Primary FSGS similarly exhibited a strong inverse correlation (*ρ* = −0.83, *p* < 0.001) (Figure 3C), indicating preservation of the compensatory globulin response. However, in contrast to MCD, the relative A/G ratio remained higher (Figure 1B), implying that differences in serum protein profiles are unlikely to reflect impaired hepatic synthesis. Rather, they may be attributable to differences in glomerular permeability and protein handling.

## 4. Discussion

Our findings indicate that SPEP reflects distinct patterns that may correlate with the specific pathomechanisms underlying different podocytopathies. By integrating clinical and electrophoretic data, two principal biological divergences emerged. First, the character of secondary FSGS protein metabolism differs significantly from that of immune-mediated podocytopathies [21]. Second, within immune-mediated forms, differences between MCD and primary FSGS become evident primarily in the setting of severe nephrotic syndrome, where the A/G ratio captures differences in the preserved glomerular size-selectivity of MCD versus the non-selective protein loss of primary FSGS.

### 4.1. Secondary FSGS: A Distinct Metabolic Pattern

The A/G ratio demonstrated excellent performance for identifying secondary FSGS (AUC > 0.98; Figure 2B,C). In this group, relative preservation of the A/G ratio suggests the absence of the pronounced electrophoretic alterations observed in immune-mediated podocytopathies [22,23].

Correlation analysis further supported this distinction. Whereas immune-mediated forms exhibited a strong inverse association between albumin and α2-globulin fractions (*ρ* > 0.8), secondary FSGS showed a comparatively weaker relationship (*ρ* = 0.57; Figure 3D). This pattern is consistent with the concept that proteinuria in secondary FSGS, which often arises from adaptive or haemodynamic mechanisms, may not provoke the same degree of hepatic compensatory response seen in classical nephrotic states [24].

Importantly, this interpretation remains inferential and reflects associations observed in serum protein fractions rather than direct measurement of hepatic synthesis rates.

### 4.2. Severity-Dependent Discrimination: A Threshold Phenomenon

A central observation of our study is that discrimination between MCD and primary FSGS became clinically meaningful only within the severe subgroup (relative albumin fraction < 40%) [25]. This suggests that the systemic response to hypoalbuminaemia may not be linear across the entire disease spectrum [26].

One plausible explanation is a threshold phenomenon, whereby substantial reductions in plasma oncotic pressure are necessary to trigger maximal compensatory protein synthesis [27]. Inclusion of patients with milder disease activity may therefore dilute detectable differences in electrophoretic profiles [28].

The pathophysiological basis for this effect may relate to qualitative differences in proteinuria [7,29]. According to the Van’t Hoff principle, colloid oncotic pressure depends on particle number rather than mass concentration [30]. Because albumin (≈67 kDa) contributes more substantially to osmotic pressure per gram than larger globulins (e.g., IgG ≈ 150 kDa), highly selective albumin loss—as typically observed in MCD—may result in a more pronounced reduction in oncotic pressure per unit of proteinuria [31]. In contrast, the non-selective proteinuria of FSGS includes a significant fraction of higher molecular weight proteins (e.g., IgG) that exert comparatively lower osmotic influence per gram [32].

Under conditions of severe oncotic pressure reduction, these qualitative differences in protein loss may translate into distinct alterations in serum protein homeostasis [33]. In MCD, preserved size-selectivity of the glomerular barrier may favour retention of newly synthesised high-molecular-weight proteins, resulting in a pronounced decrease in the A/G ratio [34]. In primary FSGS, relative non-selectivity may permit partial loss of these larger proteins, attenuating their accumulation in serum [35].

These mechanisms remain hypothetical but are consistent with the observed restriction of diagnostic discrimination to the severe subgroup (Figure 2D).

### 4.3. Clinical Implications: A Practical Electrophoretic Signal

From a clinical perspective, two practical implications emerge. First, an A/G ratio > 1.0 strongly favours secondary FSGS over immune-mediated podocytopathies (Figure 2B,C). Second, in patients with severe nephrotic syndrome, a relatively preserved A/G ratio (>0.5) may indicate non-selective glomerular injury consistent with primary FSGS (Figure 2D). In such cases, careful histopathological evaluation—including examination of additional biopsy sections—may be warranted to avoid misclassification as MCD [36].

Importantly, these observations should be interpreted as adjunctive, not definitive, diagnostic indicators and require external validation.

## 5. Conclusions

**Figure 4:**
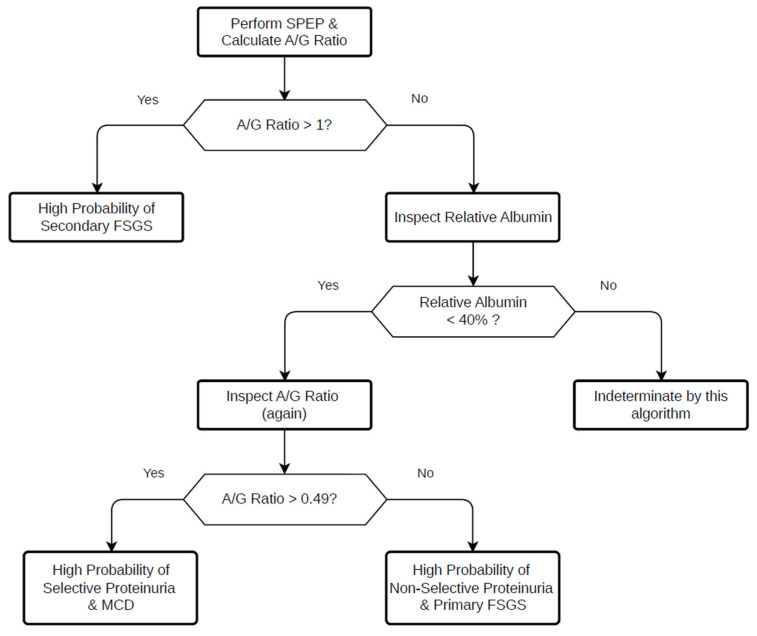
Proposed clinical flowchart for the differential diagnosis of podocytopathies using serum protein electrophoresis. The algorithm uses the albumin-to-globulin (A/G) ratio as the primary decision point. An A/G ratio > 1.0 suggests secondary FSGS. In patients with A/G < 1.0 and severe nephrotic syndrome (relative albumin fraction < 40%), an A/G threshold of 0.49 is applied to differentiate MCD from primary FSGS. The flowchart illustrates a potential non-invasive framework to support pre-biopsy diagnostic assessment.

Our findings demonstrate that serum protein electrophoresis provides clinically meaningful information in the evaluation of podocytopathies (Figure 4).

The A/G ratio shows strong discriminatory capacity for identifying secondary FSGS, where values > 1.0 strongly argue against immune-mediated disease. Within the context of severe nephrotic syndrome, a markedly reduced A/G ratio (<0.49) characterises MCD and reflects distinct differences in serum protein homeostasis compared with primary FSGS. These findings suggest that differences in glomerular selectivity contribute to divergent electrophoretic patterns once colloid oncotic pressure falls below a critical threshold.

Overall, the integration of the A/G ratio—as a simple, widely available biomarker—into clinical assessment may refine pre-biopsy probability estimates and support more targeted diagnostic strategies. Our results underscore that nephrotic syndrome is shaped not only by the magnitude of protein loss but also by its composition and the subsequent compensatory response. Further studies are required to validate these observations in independent cohorts and to elucidate the underlying mechanistic pathways.

## Figures and Tables

**Table 1:** Baseline demographic and laboratory characteristics of patients with biopsy-proven podocytopathies.

Variable	MCD (*n* = 27)	Primary FSGS (*n* = 27)	Secondary FSGS (*n* = 20)	*p*-Value
Demographics				
Age (years), mean ± SD	54.7 ± 16.3	51.9 ± 15.7	45.7 ± 13.3	0.114
Gender (Female), *n* (%)	16 (59.3%)	14 (51.9%)	7 (35.0%)	0.251
Laboratory Data				
Serum Albumin (%), median (IQR)	36.5 (30.4–42.6)	37.3 (35.0–48.0)	60.3 (58.1–63.3)	<0.001
α1-globulin (%), median (IQR)	3.9 (3.4–4.8)	4.1 (3.7–4.5)	2.8 (2.3–3.2)	<0.001
α2-globulin (%), median (IQR)	27.3 (21.9–33.0)	23.8 (19.8–27.7)	11.3 (10.8–12.1)	<0.001
β-globulin (%), median (IQR)	21.4 (19.0–23.9)	18.7 (16.7–23.1)	13.4 (12.3–14.0)	<0.001
γ-globulin (%), median (IQR)	9.8 (8.3–12.0)	10.8 (9.0–12.4)	11.1 (9.9–12.6)	0.263
A/G Ratio, median (IQR)	0.58 (0.44–0.74)	0.60 (0.54–0.92)	1.52 (1.39–1.73)	<0.001

## Data Availability

The data presented in this study are available on request from the corresponding author.

## Abbreviations

The following abbreviations are used in this manuscript:

A/G	Albumin-to-Globulin
ANOVA	Analysis of Variance
AUC	Area Under the Curve
FSGS	Focal Segmental Glomerulosclerosis
HSD	Honestly Significant Difference
IQR	Interquartile Range
KDIGO	Kidney Disease: Improving Global Outcomes
MCD	Minimal Change Disease
ROC	Receiver Operating Characteristic
SD	Standard Deviation
SPEP	Serum Protein Electrophoresis
